# Pennsylvania

**Published:** 1896-05

**Authors:** 


*Pennsylvania.* Excerpt from the advance sheets of the report of State Veterinarian Pearson:

*Tuberculosis.* Perhaps no subject connected with the health of animals has received so much attention in the agricultural press during the past two or three years as this disease. For a long time tuberculosis was regarded as an infection inherited or caused by bad surroundings. At the present time the proof is conclusive that it is an infectious disease, and that it develops only upon exposure to a tuberculous animal or to its products. The active cause of the disease is a bacillus which was discovered by Koch. The germs are contained in the excretions and dejecta of tuberculous animals, and when they are carried into the body of a healthy animal in sufficient numbers they set up the disease. They are contained abundantly in the material coming from diseased organs. Anything that reduces the vitality of the animal renders it more liable, upon exposure, to contract this disease. So that close stabling, but little exercise, irrational feeding, bad ventilation, etc., while they do not cause tuberculosis, render it much easier for the disease to develop in an exposed animal.

When this contagious disease appears in a herd, it is necessary, if we wish to exterminate it, to remove all of the diseased animals. In the past this was a matter of the greatest difficulty, because the symptoms are so indefinite, in many cases, that the disease cannot be recognized even when the animal is in such a condition that it is dangerous to its associates. This difficulty has been overcome during the past few years by the use of tuberculin, a substance made from artificial cultures of the bacilli of tuberculosis. When a small amount of this substance is injected beneath the skin of a tuberculous animal a rise of temperature occurs within from six to sixteen hours, while if the animal is free from tuberculosis there is no unusual elevation of temperature. While this means of diagnosis is not absolutely infallible, it can be relied upon, when properly carried out, in nearly every case, and is the most accurate known method for diagnosing the disease. By the use of the tuberculin-test it is possible to discover and remove all of the infected animals in a herd; it only remains, then, to thoroughly disinfect the premises, improve the sanitary conditions, and prevent the introduction of other tuberculous animals, to keep the herd free from this disease. A second examination should always be made within six months or a year after the first one, in order to discover possible cases that were overlooked during, or that developed after, the first test. It has been shown by the experiments of Bang, in Denmark, and of a number of veterinarians in this country, that if this work has been done thoroughly, in the way indicated, the disease can be completely eradicated in herds.

Bovine tuberculosis is of extreme importance in so far as the health of cattle is concerned, because it is a more or less rapidly spreading, contagious, and fatal disease. But it is of still wider public importance because it may be contracted by people through consuming milk that contains bacilli of the disease. On account of this fact, the consumption of milk has been somewhat restricted and the market for milk and dairy products contracted. This has led the authorities of some States to adopt radical exterminative measures in combating this disease. The New England States have active commissions working under special laws and receiving appropriations for the purpose of inspecting herds and destroying tuberculous animals. In all cases some remuneration is given the owner to at least partly reimburse his loss. In Massachusetts the appropriations for this purpose amount to $185,000. In New York this matter is looked after by a special committee of the State Board of Health. During the past year they have followed methods similar to those in operation in parts of New England. Quarantine measures have been adopted in some of the New England States, whereby it is necessary that all animals brought in from another State must be accompanied by a certificate from a veterinarian, based upon the tuberculin-test, showing freedom from tuberculosis, or submit to an examination with tuberculin at the point at which they enter the State. A law has been enacted in France during the past autumn requiring the examination with tuberculin of every herd in which there is reason to think that tuberculosis exists, and all tuberculous animals are quarantined or killed.

*Sudden Deaths.* It happens occasionally that a number of cattle die suddenly from some unknown cause. The probable cause of these deaths, in many cases, is one of the diseases named above, but it may be that there are some diseases of which but little is known, or that certain poisons have operated to destroy the animals. It would be of great value to a proper understanding of such cases if they could be reported in as great detail as is possible to this office. Such reports might lead to important developments.

General sanitary measures seem yet to be the only safeguards in limiting the losses from hog-cholera and swine-plague. Cremation of the bodies of hogs dying from these diseases is the only true safeguard, while careless burial, throwing the carcasses in water-courses, is sure to perpetuate its existence and disseminate losses over widespread territory.

As to trichinosis, thorough cooking of all pork products is all that is necessary.

The sheep of Pennsylvania are very free from the ravages of scab, foot-rot, and parasitic diseases, so much more common in certain Western breeding districts.

The need of great care in the diagnosis of rabies, and the thorough study of suspected cases, and the resorting to experiments to determine whether the animal suffered from the same after the biting of others, is strongly urged.

The need of better methods for the lessening of great losses among the poultry interests from contagious and infectious diseases, so prevalent among them, is urged, and the wisdom of better informing the people of the State is suggested.

